# Light and Darkness: Prevalence of Hepatitis E Virus Infection among the General Population

**DOI:** 10.1155/2014/481016

**Published:** 2014-02-10

**Authors:** José-Manuel Echevarría

**Affiliations:** Department of Virology, National Centre of Microbiology, Instituto de Salud Carlos III, Road Majadahonda-Pozuelo, Km2, Majadahonda, 28220 Madrid, Spain

## Abstract

Human hepatitis E virus (HHEV) spreads early in life among the population in areas endemic for genotype 1 and infects mainly adults in areas endemic for genotype 3, where it would be responsible for about 10% of cases of suspected acute viral hepatitis of unknown etiology and for a number of subclinical, unrecognized infections. The overall prevalence of antibody to HHEV is high in most of the former areas and low in most of the later ones, but wide regional differences have been recorded in both cases. “Hot spots” of HHEV infection would exist for both types of strains in particular regions or among particular populations of the world. Studies on pork derivatives, shellfish bivalves, and vegetables for HHEV contamination at the sale point need to be extended for evaluating the impact of the agent on food safety, and the meaning of the finding of HHEV genotype 1 genomes in urban sewage from developed countries should be established through active surveillance. Consensus about technical issues in regard to anti-HEV testing would improve the knowledge of the HHEV epidemiology. Studies in particular regions and populations, and introduction of molecular diagnosis in the clinical setting as a routine tool, would also be required.

## 1. Introduction

One of the mysterious aspects of hepatitis E virus (HEV) is the high seroprevalence of antibody to HEV (anti-HEV) IgG in developed countries where the infection is not endemic, despite the seldom reported cases of acute clinical hepatitis caused by HEV in these countries.

This sentence opened, five years ago, a review article on the hepatitis E virus (HEV) seroprevalence in developed countries, a matter of mystery for the authors of the review [1]. Though the infection by zoonotic HEV strains is actually endemic in these regions, there are still reasons, five years later, to share with them some perception of mystery from analyzing the data available about the prevalence of antibody to HEV (anti-HEV) in the different populations of the world. Such reasons arise both from conceptual and technical issues and from the data and set both light and darkness on the epidemiology of HEV. Next pages will try to show the enlighten areas and to suggest ways for illuminating the dark ones.

## 2. Taxonomic Status and General Proprieties of Human HEV

The family Hepeviridae includes at present five separate groups of viruses of vertebrates. Genomes from strains found among bats are the closest to the avian viruses [2]. Viruses from ferrets and rats cluster separately from human-related viruses in phylogenetic trees. Finally, strains isolated from trout draw a group independent from the remainder [3]. In a recent review of the information available, a future classification of the family into two genera was proposed on the basis of these genetic relationships, with the avian and mammal viruses drawing a single genus and the viruses from fish a second one [4]. The former genus would consist of four separate species: avian HEV, bat HEV, HEV from rodent and ferret, and human-related HEV (HHEV). This last species would be further subdivided into six genotypes, two of them found among wild boars only. Therefore, all HEV strains found among humans would belong to a single viral species consisting of four separate genotypes. Some of these genotypes are exclusive of the human beings and some are shared with other mammal species, as explained below.

The HHEV virion is a spherical-shaped particle about 30 nm in diameter whose structure resembles the structure of the calicivirus particle under the electron microscope. The viral core protein is the single structural protein of the virion but arranges in different ways to generate a series of structural units. The genome consists of a single linear species of single-stranded, 3′-capped RNA of positive polarity and of 7.3 kilobases (kb) in length which is organized in three open reading frames (ORFs) [11]. ORF1 extends for 5.1 kb and encodes at least four functional, nonstructural proteins displaying activities of methyl-transferase, protease, helicase, and RNA-dependent RNA polymerase. ORF2 encodes the core protein, which builds the capsid of the virion and is responsible for attachment and entry into the host cell and for the main stimulation of the specific immune response. ORF3 encodes a small, antigenic phosphoprotein of unknown function. HHEV is difficult to replicate in cell culture to a high titer, and laboratory assays for specific antibody testing are commonly developed with different recombinant antigens from the core protein, though some include also recombinant antigens from the ORF3-encoded protein. Assays for molecular diagnosis are usually based on amplification of sequences from the ORF1 region. Sequencing of the products may render the identification of the HHEV genotype present in the sample, but further characterization requires amplification of sequences from the ORF2 region or better full genome sequencing.

Though comparative analysis of genomes is winning an increasing relevance in biological taxonomy, other meaningful considerations must also be taken in mind for classifying viruses. In addition to the traditional criteria of virion morphology and antigenic proprieties, the survival strategy represents a trait intimately linked to the evolution of virus populations that may involve important epidemiological consequences. In the particular case of HHEV, this trait is relevant and should not be ignored by taxonomists.

## 3. Is HHEV a Single Virus?

Cross-neutralization gave herpes simplex virus (HSV) the former consideration of a single virus species consisting of two types. After some years, HSV types 1 and 2 (HSV1, HSV2) were classified into two separate species sharing most neutralizing epitopes, but lacking cross-protective immunity. Such distinction recognized, among other differential features, the radically different survival strategies of these two agents, which determine a totally different epidemiology. HSV1 spreads widely among the population very early in life and is transmitted mainly by the respiratory route among children. In contrast, HSV2 is a sexually transmitted agent whose spreading among adolescents and adults depends on the sexual behavior. Anti-HSV1 prevalence is almost uniformly high everywhere. The prevalence of anti-HSV2 is lower and displays wide differences between regions, but also in regard to the subset of population considered within a given region. Formerly, performance of antibody surveys specific for each of these two viruses was impaired by an important technical limitation. Detection of type-specific antibody required the measurement of the kinetics of the neutralization observed after mixing the sample with carefully titrated preparations of infectious virus from each type, a cumbersome approach almost unaffordable for testing hundred or thousand samples. Later on, novel and friendlier type specific tests were developed and these antibody surveys could be achieved. In a study from Spain, almost 80% of an adult female population tested positive for anti-HSV1 by immunoblot, but only 3.5% tested positive for anti-HSV2 [12]. In the USA, a study performed at the national level found 18% of white adult women positive for anti-HSV2, but the rate increased to 46% among black women [13]. Therefore, both significant regional and population-based differences existed in the epidemiology of the HSV2 infection, and such knowledge was relevant for improving the prevention of genital herpes and of the devastating neonatal disease resulting from mother-to-child virus transmission.

The present taxonomic status of HHEV as a single viral species reminds the former status of HSV. No evidence of cross-protective immunity between the different HHEV genotypes exists, and they can also be meaningfully grouped into two groups in regard to their survival strategies and their epidemiological features. Genotypes 1 and 2 (HHEV1, HHEV2) are exclusive of human beings, though HHEV1 may accidentally or artificially infect other species. They are restricted to particular geographical areas and spread often among the population as waterborne, open epidemic outbreaks. In contrast, genotypes 3 and 4 (HHEV3, HHEV4) are adapted to mammals from different orders, from swine and deer to mongoose and rat, and are distributed worldwide and infect humans sporadically through zoonotic transmission or by consumption of contaminated aliments. These behaviors most likely reflect two sharply separated evolutionary lineages, representing two different survival strategies that evolved independently since long time ago. From an evolutionary point of view wider than the strict consideration of the genetic relationships, these two HHEV lineages would perhaps merit the consideration of two different viral species, and such consideration would fit the epidemiological findings.

Diseases caused by these HHEV genotypes share identical clinical features between them and with other acute viral hepatitis. But for the high mortality recorded sometimes among pregnant women infected by HHEV1, hepatitis E can be indeed taken by clinicians as hepatitis A in the regions of the World where hepatitis A virus (HAV) remains endemic. This lack of specific symptoms and signs is characteristic of acute viral hepatitis in general, and the disease is classified as A, B, C, D, E, or non-A–E after performing laboratory studies. If HEV was divided into two species, acute hepatitis E would also be divided into two different diseases, and virologists would learn that they should try to separate them in the laboratory as they do for the rest.

The investigation of the HEV epidemiology is, therefore, influenced by these conceptual issues, since we are actually mixing two different viral infections and two different diseases into the same pot. In addition, testing samples for anti-HEV with epidemiological purposes is limited by the lack of genotype-specific assays, a limitation that researchers of the HSV epidemiology suffered from for many years in the past. Development of genotype-specific tests would, therefore, be an important requirement to enlighten the mysteries of the anti-HEV prevalence in the future.

## 4. Hepatitis E: Infection and Disease

The actual rate between disease and infection is unknown for HHEV, but the general thought is that asymptomatic infection is a common event [14]. Most diagnosis was achieved among patients suffering icteric hepatitis, and most data regarding other symptoms and signs came from the study of epidemic outbreaks due to HHEV1. In addition to jaundice, anorexia, abdominal pain, and hepatomegaly were consistently found among patients from the main studies, other symptoms like fever, nausea, or vomiting were less frequent. Fulminant hepatic failure was uncommon but in pregnant women, and the case fatality rate ranged from 0.5 to 4% among patients requiring hospitalization, who are likely a small minority. Persistent viral infection was never reported in these studies.

Clinical data from patients with acute hepatitis infected with HHEV3 are scarcer. Most cases reported were icteric and displayed elevated ALT levels in serum, very much like cases of acute hepatitis A when the comparison was performed [15]. Jaundice and biochemical alterations were more pronounced among cases confirmed by detection of viremia, and the duration of the disease was also longer among them than among patients testing negative for viral RNA in serum. However, these differences might just reflect an earlier sampling among the former. Complications were not frequent, and fatal outcome was always associated to unnoticed, prior alcoholic cirrhosis.

A careful investigation performed on people involved in an outbreak of HHEV3 infection among the passengers of a cruise ship found 11 cases of acute hepatitis and 22 asymptomatic infections (rate up to 33%) [16]. Disease was significantly associated with excessive alcohol intake, and anorexia, malaise, nausea, and dark urine were the most frequent symptoms and signs. Jaundice was observed in seven cases. Liver enzyme levels were often high among the patients but were always normal among the people experiencing asymptomatic infections. A common source of infection could not be identified for the outbreak, but shellfish intake was likely involved. In agreement with the usual male to female rate found in collections of sporadic cases of acute hepatitis due to HHEV3, the likelihood of infection was significantly higher among men than among women.

Though hepatitis E is always a self-limited disease among immune competent patients, HHEV3 persistency resulting in chronic hepatitis has been reported among patients with immune impairment [17]. Reports included transplant recipients, haematological patients receiving chemotherapy, and patients infected by human immunodeficiency virus. Rapid progression to liver cirrhosis was often observed. Ribavirin therapy and lowering immunosuppression lead efficiently to virus clearance in most cases.

In summary, hepatitis E is a mild disease that may present both as epidemics and sporadic cases. Symptoms and sings resemble hepatitis A, and the rate of asymptomatic infection might be as high as two additional infections for each case of disease. Otherwise than acute liver failure among pregnant women infected by HHEV1, complications are infrequent. Viral persistency is not rare among immunosuppressed patients and may result in rapid progression to cirrhosis, but the outcome can be efficiently prevented by the antiviral therapy.

## 5. Considerations about the Frequency and the Acquisition of the Disease

Hepatitis E is the most frequent acute viral hepatitis in developing countries, and is recorded both as sporadic cases and as epidemic outbreaks in these settings [18]. It is caused by HHEV1 in Southern and Central Asia, the Far East, and the Caribbean and by HHEV1 and -2 in Africa. Most cases from outbreaks are recorded among children. Sporadic hepatitis E caused by HHEV3 or -4 is also common in China, Japan, Korea, and Taiwan, and HHEV4 has been detected among swine livestock from India and Indonesia [18]. Molecular testing is still uncommon in clinical laboratories worldwide, and the genotype responsible for the disease is rarely identified. Therefore, a participation of HHEV3 and -4 in sporadic cases at India and South-east Asia cannot be ruled out.

Though HHEV4 may cause locally acquired hepatitis E in Western Europe, HHEV3 is responsible for the majority of autochthonous cases recorded in the continent [19]. In recent studies, HHEV was involved in 4.4% of 1027 cases of acute hepatitis tested in The Netherlands for three years [20]; 3.3% of 838 cases tested in Southwest England for two years [15]; 10.5% of 97 cases tested in Finland for nine years [21]; 9.6% of 1203 cases tested in Hungary for six years [22]; 10.8% of 277 cases tested in Spain for six years [23]; and, exceptionally, 55.7% of 52 cases tested in Italy for seven years [24]. Imported infections by HEV1 predominated in studies from Finland and, in special, from Italy. Locally acquired infections due to HEV3 were the most frequent in the remaining series. These local infections were more common among men aged over 40 than among women or among younger men and were almost never found among children and adolescents. In addition, some studies recorded regional differences in frequency rates within a given country, though the data must be confirmed by population-based, prospective studies before these differences can be accepted as a matter of fact. Updated data from Spain suggest that the prevalence of locally acquired hepatitis E could be up to 20-fold higher in the northern regions of the country than in the south, increasing gradually from south to north (author's unpublished observations).

A report from the Centers for Disease Control and Prevention recorded 26 confirmed cases of hepatitis E among 154 US patients tested in a period of seven years [25]. Fifteen patients did not travel abroad recently. Thus, the frequency of locally acquired hepatitis E in the series was 9.7%. Genotyping was performed in eight of the 15 cases, and HHEV3 was found in all samples. Communications reporting autochthonous cases of hepatitis E due to HHEV3 in other American countries were previously published from Argentina, Bolivia, Brazil, Mexico, Venezuela, and Uruguay [26–31]. Frequency of HHEV diagnosis among patients with locally acquired acute hepatitis of unknown origin ranged from 1.6% in Brazil to 30% in Venezuela, though HHEV1 strains were also detected in the last country. HHEV1 was the only genotype responsible for sporadic cases and epidemic outbreaks of hepatitis E in Cuba [32] and has been recently involved in an autochthonous case presented in Uruguay (Arbiza J, personal communication).

HHEV would, therefore, respond to about 10% of cases of non-A–D suspected acute viral hepatitis presenting in western countries among nontravelers, and collecting a significant number of cases has taken years of work from the authors of these reports. Since results from different investigations suggest a likely involvement of the consumption of pork derivatives, shellfish bivalves, or crude vegetables in virus transmission [33–36], autochthonous hepatitis E is considered at present a food-borne, low-incidence zoonosis in Europe, North America and the South Cone of this continent, in addition to an imported disease. Such consideration could be likely extended to other temperate regions such Australia or New Zealand where specific data are still scarce.

## 6. Laboratory Diagnosis of the Acute Infection

The dynamics of virus shedding, viremia, and antibody response in the acute HHEV infection displays the usual events in other systemic viral infections acquired by the fecal-oral route. The antibody window period extends for two weeks, and viremia persists at detectable levels for a variable period of time after the window closing. Virus is shed in stool from the beginning of this period, and fecal shedding persists for several weeks after the viremia is cleared. Molecular testing of stool samples provides, therefore, an excellent chance for laboratory diagnosis, but it is seldom performed at clinical laboratories because of technical issues regarding the extraction of viral RNA from stool, which requires experience for a suitable yield.

Viremia levels are lower than in other viral acute hepatitis, with the yield of serum testing being highly dependent of the analytical sensitivity of the assay used. Since commercial tests for HHEV genome detection are still scarce in the market, most laboratories performing molecular diagnosis are testing samples by in-house conventional techniques of RNA amplification by reverse transcription, polymerase chain reaction (RT-PCR). However, commercial, sensitive methods of real-time PCR will likely replace them in the short term. Retrospective testing of samples after long storage may reduce significantly the yield of molecular testing because of viral RNA degradation, which underlines that clinical laboratories must always test fresh samples.

Early serological diagnosis is based on detection of the anti-HHEV IgM response, which precedes in most cases the rising of anti-HHEV IgG. Testing is performed by indirect enzyme-immunoassay (EIA) using recombinant antigens from the core protein. Most commercial tests use antigens matching HHEV1 sequences, which are thought less sensitive for diagnosing infections caused by the remaining genotypes. However, such thoughts are not always fully supported by data. Like in other viral infections, samples reactive in EIA testing can be retested by recombinant immunoblot (RIB) for confirmatory purposes. A commercial test is available for such confirmation and includes recombinant antigens corresponding to HHEV1 and -3 core and ORF3 protein sequences. Confirmed reactivity for anti-HHEV IgM is considered diagnostic for acute infection. Since a segment of the healthy population from any geographical region of the World considered displays residual anti-HHEV IgG in serum reflecting a prior contact with the virus, the finding of this marker alone has no diagnostic value unless seroconversion is demonstrated after testing serial samples from the patient.

In agreement with these considerations, diagnostic criteria for acute HHEV infection among patients with acute hepatitis would be as follows: (1) IgM negative, RNA positive (window period); (2) IgM positive, RNA positive (early seroconversion stage); (3) IgM positive, RNA negative (post-seroconversion stage); and (4) Seroconversion to IgG antibody on follow-up. When specific IgM is the only diagnostic marker found, exclusion of acute primary infection by human cytomegalovirus and Epstein-Barr virus by specific testing is suitable, because these agents may rise up a false diagnosis of hepatitis E through polyclonal stimulation of HHEV-specific B cell subsets.

## 7. Technical Issues Would Influence Anti-HHEV IgG Testing

Though the data available support the reasonable robustness of the current assays for anti-HHEV IgM testing in the diagnosis of acute hepatitis E, doubts about the specificity and/or sensitivity of these reagents in recognizing residual immunity to HHEV among the general population by anti-HHEV IgG testing emerged in the last years. On one hand, testing enzyme immunoassay (EIA-) reactive samples by recombinant immunoblot (RIB) classified 28–50% of them as anti-HHEV negative if RIB was used as a confirmatory test [8, 20, 37]. On the other hand, comparison of the performance of current EIA assays using a standard preparation of anti-HHEV showed considerable differences that were claimed to reflect the insensitivity of most of them, leading to suggest that results from all seroprevalence studies on HHEV performed in the World would have largely underestimated the reality [38].

In a report from France, a new testing of samples by the test claimed the most sensitive one rose up by three times the prevalence of anti-HHEV found formerly among the blood donors from Toulouse (16.6 versus 52.5%) [39]. The very same discrepancy between this particular test and another one was observed among a group of samples from transplant recipients from Marseille two years later (10.9 versus 31.3%) [40]. Aiming at knowing whether the apparently more sensitive test (Wantai, China) was actually more sensitive or less specific than the other one (Adaltis, Italy), the authors of this last study tested by RIB six samples reacting in both assays and 14 reacting only in Wantai. These six samples and 10 of the last 14 samples tested positive, but the remaining four samples tested negative. If any conclusion can be drawn from the data, it would either be that the Wantai test provides false-positive results for anti-HEV IgG or that the RIB test is not satisfactory for the purpose of confirmation. It was certainly shown, however, that the Adaltis test was rather insensitive, but this would only affect the results from seroprevalence studies performed with this particular reagent.

There are, therefore, reasons to pursuit efforts in standardizing the HHEV serology and to agree about collaborative studies which can lead to a consensus about the technology suitable for performing serosurveys and about the validity of the data collected during the last two decades in the different regions. Until these objectives are achieved, what can be done is just to look at these data and try to understand what they may mean.

## 8. A Look on the Data

Tables 1, 2, 3, and 4 summarize the results reported from studies involving collections of samples more or less representative of the general population of countries or regions from Europe, South Asia, the Far East, the Middle East and the Americas.

South Asia and the Far East displayed often high prevalence rates. India, Malaysia, and Southern China displayed the highest rates among children (up to 20–50%). Rates higher than 50% were found also among adults from Hong Kong and other regions of China, and low rates (less than 10% among adults) were consistently reported from Japan. This would roughly match the impact of HHEV1 (and of subtypes 1a and 1b in particular) on epidemic hepatitis E. HHEV1, -3 and -4 overlap, however, widely in the region and rates higher than reported would have been expected from countries like Thailand, Indonesia, or Vietnam.

In the Middle East, the prevalence was low everywhere but in Egypt. With this single exception, anti-HHEV was almost absent among children and did not reach 20% among adults. However, most reports are from Turkey and Iran, which can be considered countries of low endemicity for HHEV.

In regard to Europe and the Americas, the rates reported were low and pretty much the same for all studies but for three performed in the UK, the US, and Bolivia. In the UK, the prevalence was sixfold lower among the adult population of London sampled in 1988-89 than among the population of England and Wales sampled throughout 1991 and 2004 (3.9 versus 27%) [9, 41]. In the US, anti-HHEV rose with age up to 45% among adult men aged over 60 years in a survey performed by the National Institutes of Health and the Centers for Disease Control at the national level. The rate among adults would have been expected higher in areas close to the Mexican border than in the rest of the country, but the data available show actually the very opposite (1.6 versus 42%) [10, 42]. Though Mexico is usually included in the list of highly endemic countries for hepatitis E, this is just based on the report of an epidemics developing 26 years ago that was attributed to a unique HHEV2 strain (genotype 2a) [43] never found again. As for other areas of Latin America formerly thought as highly endemic for HHEV, the data available, or better the scarcity of them in the case of Mexico, do not support the assertion [44].

As a complement to these data, Table 5 summarizes the results from representative studies performed among blood donors worldwide. Most of them agree with the data from the general population of adults, and significant discrepancies between studies performed within a given country are again noticed. In the US, results from the two studies available would suggest that the prevalence of anti-HHEV is 13-fold higher in Washington DC than in Northern California [45, 46]. In the United Kingdom, donors from Bristol displayed a prevalence almost fourfold higher than the one found recently among Scottish donors [38, 47]. In France, the prevalence would be more than 16-fold higher at Midi Pyrénées than at Île de France or Pays de Loire [39, 48]. Differences might respond in some cases, but not always, to the technical issues discussed above.

**Figure 1:**
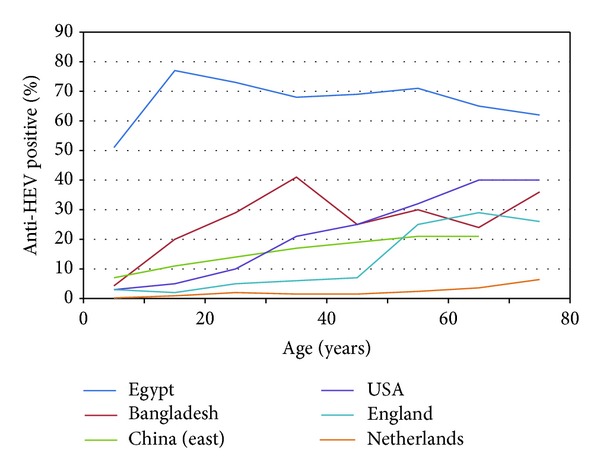
Examples of the patterns of acquisition of anti-HHEV with age among the population from regions endemic for HHEV1 (Egypt, Bangladesh, and East China) [5–7] or HHEV3 (USA, England, and The Netherlands) [8–10].

Comparison of data shows that the greatest regional differences are seen among children and indicates that HHEV spreads earlier in life among the population of Asia and Egypt than of the rest of the World (Table 6). Among the adults, the differences do not look so sharp when only the ranges are considered. Independently of the overall prevalence, anti-HHEV is acquired earliest in life in regions endemic for HHEV1 in comparison with the regions endemic for HHEV3 (Figure 1). However, the prevalence of anti-HHEV reported for the oldest population groups was almost the same in the US than in Bangladesh and was lower in East China than in England. The low-prevalence pattern found in The Netherlands is representative for other Western European countries like Spain or San Marino but also for Asiatic countries like Japan where both HHEV1 and HHEV3 autochthonous infections are reported.

The significance of this overview is, however, limited by the finding of some very significant variations of the anti-HHEV prevalence when different populations from a single country or region are compared (Table 7). Particular ethnic groups and some rural populations of South Asia and of the Far and the Middle East seem to represent true “hot spots” of the HHEV epidemiology. Just Egypt would constitute such a hot spot as a full country, since the prevalence keeps high among rural populations from both the Lower and the Upper Nile River. Whether this fact is characteristic of the Egyptian rural setting or is also shared by the population from great cities like Cairo or Alexandria is unknown.

In Latin America [44], the rate of anti-HHEV found 15 years ago among homeless children from Cochabamba (66%) [49] remains more than threefold higher than the highest ever reported for any other population group in the region. However, reagents used to perform the study were primitive, and further studies are required before qualifying this region of Bolivia as highly endemic for HHEV. Rates recorded in the Bolivian Amazon in the same report (up to 26% among the Yurakare Amerindians) were also higher than the rates found before among Amerindian populations from tropical forests of Venezuela outside the Amazon (5.4 and 9.7%) [50, 51]. New data obtained with updated reagents will eventually enlighten the epidemiology of HHEV in the tropical woodlands of South America and confirm the differences they may display.

The anti-HHEV prevalence rate reported for blood donors aged 58 to 65 years from the French region of Midi Pyrénées (70%) [39] is a single and unexpected European spot in Table 6. It seems unlikely that a rate of 2.5 to 4-fold higher than the highest age-specific rate ever reported in Europe may merely respond to technical issues, and it adds reasons to think that the southwest of France might also be a “hot spot” of the HHEV epidemiology. The existence of particular regions displaying a comparatively high incidence of HHEV infections has been also suggested for other European countries [52, 53].

## 9. Sources of HHEV and Routes of Transmission

Sources for new human infections by HHEV1 and -2 should always be infected people shedding the virus in stool for a short period of time during the acute, self-limited infection, since no animal reservoir has yet been consistently demonstrated for these genotypes. Drinking water would be the main vehicle for transmission, and crude vegetables and shellfish bivalves contaminated by sewage would play some role. Hepatitis E due to these viral genotypes is, therefore, epidemiologically similar to hepatitis A, and the lesser stability of the infectious HHEV particle [54–56] would explain why the disease is no longer present in settings of high sanitation standards but from importation. Surveys performing comparison of anti-HHEV and anti-HAV prevalence in countries endemic for HHEV1 showed that the former spreads among the population much less than the second one and suggested that the lesser stability of the HHEV particle matters very much for the epidemiology [5, 57–61]. Cases of HHEV1 infection secondary to importation have not yet been reported from European countries. However, HHEV1 RNA has been reported twice from sewage samples from the city of Barcelona, which might allow the contamination of shellfish bivalves and lead eventually to the local acquisition of HHEV1 infection by consumption of seafood, as suspected for a small outbreak of HHEV4 infection reported from Italy [36]. Whether the detection of HEV RNA in sewage reflects always and everywhere the presence of infectious viral particles is unknown.

HHEV1 strains involved in locally acquired hepatitis E in Latin America are highly related and are also genetically close to some strains circulating in India. These findings suggest a more or less recent episode of secondary spread after importation. Reporting of outbreaks of acute hepatitis involving dual infections by HHEV1 and HAV from Caribbean countries (Cuba, Venezuela) would, in addition, mean that such episodes may result in naturalization of the imported strains when the sanitation conditions are favorable for the spread of the virus among the population. Extending studies about the circulation of HHEV1 in Uruguay and in the neighbor, temperate countries of the South Cone of South America, and investigation of viral strains responsible for the high anti-HHEV prevalence reported among the members of some isolated Amerindian communities from the Amazon basin, would enlighten the origin and the role of this epidemic genotype in the continent.

Main virus sources and routes of transmission are, however, less known for human HHEV3 and -4 infections. Studies involving investigation of risk factors on a significant number of patients with locally acquired HHEV3 infection and a control group have been reported only from Germany [62]. Among 45 patients studied, consumption of raw or undercooked beef and wild boar meat, pig offal, or pig internal organs other than liver were the only factors that could be recorded in at least 20% of cases with an OR >2 in comparison with controls. Patients reported from England and Wales did not, however, share these features with the German patients [63]. In addition, no significant risk factors common to at least 50% of these German patients were found. Consumption of raw or undercooked pork products is commonly thought as a relevant risk factor for acquisition of HHEV in Europe. However, it was almost as common among the German patients as among the matched controls (78.6 versus 66.4%; OR = 2.0) and was not recognized by any of the 28 British patients investigated. The link between pork meat consumption and acquisition of hepatitis E is not, therefore, so clearly established as it has been often stated in the literature.

Pork derivatives include sausage, and this is a single English word for describing a wide diversity of products prepared by Europeans in many different ways, from air or smoke-drying to boiling and cooking. They enjoy a wide range of specific names in other European languages, which should be taken into account for a proper identification. Consumption of air-dried pork derivatives (i.e., Spanish serrano ham, chorizo and salchichón, Italian prosciutto, etc.) is traditional in Spain and Italy and is likely much more common than in any other region of the World. Hepatitis E is, however, not especially frequent in these countries and the prevalence of anti-HHEV among the population is lower in them than in England or Germany (2.1–7.3% versus 3.9–27%, see Table 1). If sausage was involved in HHEV3 transmission, it remains, therefore, to be identified what kind of sausage is relevant and what is not, since the procedure followed for preparation might perhaps matter a lot when infectious virus is present in the pork meat at the beginning of the process. The finding of HHEV3 genome in some unidentified kind of sausage purchased at a few sale points in Spain [35] suggests that extending studies in aliments would be important to understand better the epidemiology of HHEV in developed countries.

In summary, improving the knowledge about the sources and routes of transmission of HHEV will require a multidisciplinary strategy. Specialists in public health virology, epidemiology, veterinary medicine, environmental health, and alimentary safety should coordinate research efforts and share information in order to draw the full picture of the problem.

## 10. Light and Darkness

HHEV1 shares with HAV many epidemiological similarities, but is less prevalent among the population because of the lesser stability of its particle. Since the opportunity of becoming infected by HHEV1 is lesser than by HAV, the prevalence of anti-HHEV increases more slowly with age, and primary infections among adults are more common. These circumstances explain why HHEV1 became just an imported agent in the developed World and may also explain regional and population-based differences of the prevalence in endemic areas. It should be expected that improvement of the sanitation of drinking water and vegetables will help the control of HHEV1 in a shorter time than of HAV in these areas, but cocirculation of HHEV3 and -4, which are thought less prevalent, will interfere the evaluation of the impact of these improvements unless genotype-specific diagnosis of clinical cases are performed on a routine basis, and genotype-specific anti-HHEV tests can be used to perform serial, population-based surveys. Such conclusions could likely be extended to HHEV2, but the scarcity of studies about hepatitis E in Africa is a limitation. In addition, prospective studies in Mexico and neighbor countries would be required to enlighten the role that the missing subgenotype HHEV2a could play in the Americas.

There is, however, much more darkness in the epidemiology of HHEV3. On one hand, the high prevalence of anti-HHEV found among adults from some western countries like the US and France would not be at all expected from a low-incidence zoonosis transmitted by food in regions where aliments are produced and commercialized under rather strict regulations. On the other hand, the similarities displayed in Figure 1 by the curves of anti-HHEV acquisition for the oldest population groups from Bangladesh, the US, and the UK are also difficult to understand, provided that they would respond to agents of so dissimilar epidemiological properties like HHEV1 and HHEV3. A relatively high prevalence would not be contradictory with a low incidence of the disease if the acute infection was very often symptomless, as reported for HHEV3 [16], but the high prevalence seems surprising in particular areas. In the US study, the prevalence was higher at the Middle West and the west than at the south or the Northeast and did not display major differences in regard to sex or ethnicity [10]. However, it always increased slowly with age, with the rate recorded among children being low everywhere. Investigation of these likely “hot spots” of HHEV3 prevalence would be a priority for understanding the epidemiology of HHEV in temperate countries. In addition, technical issues concerning anti-HHEV testing must be clarified, and it seems likely that development of genotype-specific tests would also be of some help, if such achievement becomes possible.

## 11. Conclusion

In conclusion, the prevalence of anti-HEV in the World is no longer a matter of mystery, but some mysteries still remain to reveal. Among the 2146 articles displayed by the Pub-Med data base under the search term “hepatitis E virus” since 1990 to the time of writing this conclusion, 40% was published during the past five years, and year 2013 would likely break again the record number of 209 articles set during the past one. By following that way, mysteries will for sure become revealed sooner best than later.

## Figures and Tables

**Table 1:** Prevalence of anti-HHEV among the general population of Europe.

Country	Number of samples	Anti-HHEV rate (%)			Reference
		Children	Adults	Overall	
United Kingdom	710	—	3.9	—	[41]
	1591	2.0-3.0	5–27	13	[9]
Italy	1889	—	2.6	—	[64]
	3511	—	2.9	—	[65]
San Marino	2233	—	1.5	—	[66]
Spain	2529	4.6	7.3	6.0	[67, 68]
	2305^a^	0.5	2.1	1.1	[37]
Germany	4422^a^	—	17	—	[69]
The Netherlands	7072^a^	0–0.3	1.4–6.4	1.9	[8]

^a^Anti-HHEV screened or confirmed by RIBT.

**Table 2:** Prevalence of anti-HHEV among the general population of South Asia and the Far East.

Country	Number of samples	Anti-HHEV rate (%)			Reference
		Children	Adults	Overall	
India (north)	2070	24–29	—	—	[70]
India (Andaman)	814	13–40	16–77	15–73	[57]
India (Chennai)	185	5.3–17	—	—	[71]
India (south)	2279	0.6–8.9	9.2–36	9.1–23	[72]
Bangladesh	1134	—	23	—	[5]
Pakistan	540	14	—	—	[73]
Thailand	513	—	23	—	[74]
Malaysia	132	40–50	43–67	44–50	[75]
Indonesia	1115	—	0.5–20	—	[76]
Vietnam	646	3.0–5.0	11–19	9.0	[58]
China	8762	5.4–4.2	9.8–46	18	[59]
China (south)	3844	10–21	40–66	44	[77]
China (east)	12052	6.7–13	14–23	17	[6]
China (Fujian)	1151	—	23	—	[78]
China (Han)	7376	5.2–12	20–57	24	[79]
China (Hui)	2258	3.1–4.0	2.1–6.8	3.6	
Hong Kong	934	—	19	—	[60]
	450	6.0–8.0	18–60	28	[80]
Taiwan	984	0.3	11	4.4	[81]
	997	1.5–9.6	8.8–13	6.4–8.8	[82]
	2538	3.4	—	—	[83]
Japan	1253	—	4.6–6.7	—	[84]
	22027	—	2.7–6.6	—	[85]
Korea	147	—	14–23	—	[86]

**Table 3:** Prevalence of anti-HHEV among the general population of the Middle East.

Country	Number of samples	Anti-HHEV rate (%)			Reference
		Children	Adults	Overall	
Turkey	1374	—	5.9	—	[87]
Turkey (Istanbul)	909	2.1	—	—	[88]
Turkey (Antalya)	338	0.9	—	—	[89]
Turkey (Anatolia)	321	—	12-13	—	[90]
Turkey (Aydin)	386	—	7.0	—	[91]
Turkey (Duzce)	589	0.3	—	—	[92]
Turkey (Trace)	580	—	2.4	—	[93]
Iran (Nahavand)	304	—	9.3	—	[94]
Iran (Isfahan)	816	0.9	8.1	3.8	[95]
Iran (Sari)	1080	1.2	7.3	2.3	[96]
Iran (west)	400	—	7.8	—	[97]
Iran (Teheran)	551	—	7.9–15	—	[98]
Yemen	356	8.0	15	11	[99]
Israel	1416	—	1.8–2.8	—	[100]
Egypt	10026	36–76	48–76	68	[101]
	100	26	—	—	[102]
	2428	—	84	—	[7]

**Table 4:** Prevalence of anti-HHEV among the general population of the Americas.

Country	Number of samples	Anti-HHEV rate (%)			Reference
		Children	Adults	Overall	
USA	18695	1.0–5.0	39–42	21	[10]
USA (Texas)	864	—	0.4–1.6	—	[42]
Canada (Inuit)	393	2.6	3.1	3.0	[103]
Greenland (Inuit)	503	—	3.0	—	[104]
Venezuela (urban)	184	—	1.6	—	
Venezuela (rural)	204	—	—	3.9	[50]
Venezuela (Amerindians)	223	—	5.4	—	
	463	—	9.7	—	[51]
Nicaragua	399	—	4.6–8.0	—	[105]
Argentina	1304	0.15	—	—	[106]
Chile	168	1.2	—	—	[107]
Chile (Amerindians)	100	—	17.0	—	[108]
Bolivia (rural)	490	—	7.3	—	[109]
Bolivia (rural)	186	—	20	—	
Bolivia (urban)	193	66	31	49	[49]
Bolivia (Amazon)	318	0–14	7.0–30	20	
Mexico	3549	1.1	14	10	[110]
Brazil	1196	4.5	—	—	[111]
Cuba	209	—	5.3	—	[61]
	469	—	10.0	—	[112]

**Table 5:** Results from selected studies reporting the prevalence of anti-HHEV among blood donors in the World.

Country	Donors tested	Anti-HHEV rate (%)	Reference
USA	5000	1.3	[45]
	1939	19	[46]
Chile	1360	8.0	[113]
Argentina	2157	1.8	[114]
Cuba	1149	1.4	[115]
Brazil	996	2.3	[116]
France	1998	3.2	[48]
	512	53	[39]
Portugal	1473	2.5	[117]
Spain	863	2.8	[118]
United Kingdom	500	16	[38]
	1559	4.7	[47]
Germany	1019	6.8	[119]
China	44816	33	[120]
Japan	12600	3.4	[121]

**Table 6:** Summary of the anti-HHEV prevalence reported from different regions of the World.

Region	Anti-HHEV rate (%)			
	Children	Adults	Overall	Blood donors
Far East	0.3–21	2–75	4–44	3–33
South/southeast Asia	0.6–50	0.5–67	9–73	—
Middle East	0.3–76	2–84	2–68	—
USA/Canada	0–5	0.4–42	3–21	1–19
Latin America	0–14	0–30	10–49	1–8
Western Europe	0–5	1–27	1–13	3–53

**Table 7:** Studies reporting anti-HHEV prevalence higher than 50% among specific population groups.

Region	Population group	Anti-HHEV rate (%)	Reference
South Asia and the Far East	Orang Asli population older than 11 years (central Malaysia)	50–67	[75]
	Tribes from Andaman Islands (India)	50–100	[57]
	Guangxi rural population older than 60 years (China)	70–80	[77]
	Bangladeshi rural population older than 80 years	67	[5]
	Chinese Han older than 60 years	57	[59]
	Hong Kong population older than 80 years	52–60	[80]

Middle East	Pregnant women from the Nile Delta	84	[7]
	Lower and Upper Egyptian rural population older than four years	51–78	[101]

South America	Cochabamba city homeless children (Bolivia)	66	[49]

Western Europe	Blood donors older than 58 years from Toulouse, France	70	[39]
